# Sensor-Based Mobile App for Assessment and Tracking (SMAAT): Cross-Sectional Usability Study

**DOI:** 10.2196/98984

**Published:** 2026-08-04

**Authors:** Yury Shevchenko, Hyunjoo Hong

**Affiliations:** 1 Research Methods, Assessment, and iScience, Department of Psychology, University of Konstanz, Universitätsstraße 10, Konstanz, Baden-Wurttemberg, 78464, Germany, 49 1784188154; 2 Technology and Society Laboratory, Empa, Swiss Federal Laboratories for Materials Science and Technologies, St. Gallen, Canton of St. Gallen, Switzerland

**Keywords:** ecological momentary assessment, mobile health, smartphone, digital phenotyping, experience sampling

## Abstract

**Background:**

Smartphone-based ecological momentary assessment (EMA) is increasingly used in digital health research to capture behaviors, symptoms, and psychological states in real time and in natural environments, offering advantages over traditional retrospective measures in terms of ecological validity and reduced recall bias. Despite the potential of EMA for advancing mobile health and digital phenotyping apps, accessible technical solutions that enable researchers without software engineering expertise to design and deploy EMA studies remain limited.

**Objective:**

This study aimed to (1) describe the development and core features of a sensor-based mobile app for assessment and tracking (SMAAT), a research platform for designing and deploying mobile surveys and EMA studies, and (2) report initial usability findings from a cross-sectional study using a novel swiping response format.

**Methods:**

SMAAT consists of a web-based dashboard for researchers and companion iOS (Apple Inc) and Android (Google) apps for participants, providing a visual survey builder, multiple notification schedules (including fixed, random, interval, event-based, and geofenced prompts), gamification mechanics, and tools to monitor participant compliance. To evaluate the platform in use, we conducted a between-participants cross-sectional study in which 97 university students and 132 online panel participants completed blocks of binary questions on smartphones using either a swiping or tapping response format, followed by usability and user-experience questionnaires.

**Results:**

In the cross-sectional study, SMAAT supported successful study setup, enrollment, and survey completion across both iOS and Android devices without major technical problems, and participants in both samples completed the full protocol in a single session. Performance on the binary tasks was generally high, with swiping and tapping showing broadly comparable response-time and accuracy patterns, and no clear disadvantages for swiping or consistent effects of response orientation. Usability and pragmatic user-experience ratings were high across conditions, with no meaningful differences between swiping and tapping, while Prolific participants reported higher usability and pragmatic quality than students. Hedonic ratings descriptively favored swiping, although this difference did not reach conventional statistical significance.

**Conclusions:**

SMAAT is a flexible smartphone-based research platform for configuring and deploying mobile surveys and EMA studies with diverse item types and notification logics. Initial findings show that SMAAT can support reliable cross-sectional data collection across heterogeneous devices and samples, and that a swipe-based response format can be implemented without compromising task performance, usability, or pragmatic user experience relative to tapping.

## Introduction

### Background

#### Overview

Sensor-based mobile app for assessment and tracking (SMAAT) is a research platform that allows users to design and deploy mobile surveys and ecological momentary assessments (EMAs) via a web dashboard and companion iOS (Apple Inc) and Android (Google) apps. The platform provides a visual survey builder with a broad range of item types (including traditional types such as multiple choice or Likert scales and innovative formats such as swiping and AI chatbots), multiple notification schedules (eg, fixed, random, interval, event-based, and geofenced prompts), gamification mechanics, collection of sensor data (eg, location, accelerometer, and device motion), and tools to monitor participant compliance. In this work, we describe the development of SMAAT and report initial usability findings from a cross-sectional study using a novel swiping response format.

#### The Shift to Mobile and EMA

Research in psychology and health sciences has increasingly turned to EMA and related experience-sampling methods as alternatives to retrospective questionnaires. First systematized by Csikszentmihalyi and Larson [1] and later reviewed by Shiffman et al [2], EMA involves repeated real-time sampling of participants’ behaviors, experiences, and psychological states in their natural environments, rather than asking them to recall past states in a clinic or laboratory. The core methodological advantages include the reduction of recall bias, higher ecological validity, and the ability to capture within-person temporal dynamics and contextual associations that are systematically missed by single-occasion retrospective measures [2-6].

The widespread availability of smartphones has made EMA substantially easier to implement in large and diverse samples, because participants can receive prompts and complete short surveys on their own devices at any time and location [7]. Smartphone-based EMA eliminates the burden of carrying dedicated research hardware, and the ubiquity of iOS and Android devices allows researchers to reach populations that were previously difficult to recruit into intensive longitudinal designs. However, leveraging the full potential of smartphone EMA while maintaining high data quality and participant engagement requires platforms that are both technically robust and easy and pleasant to interact with repeatedly across the day [8].

EMA and intensive longitudinal designs have found particularly wide application in mental health and behavioral medicine research, where capturing within-person fluctuations in mood, stress, symptoms, or health behaviors is of direct clinical and scientific value. Studies using EMA have advanced understanding of depression, anxiety, chronic pain, substance use, and physical activity by documenting the dynamics of these phenomena in everyday life in ways that single-occasion assessments cannot [2,9,10]. The integration of passive smartphone sensing, such as GPS, accelerometry, or physiological signals, with active EMA surveys is increasingly central to digital phenotyping approaches that aim to derive clinically relevant indicators from real-world data [11-13]. This integration also represents a direction that platforms like SMAAT are designed to support in future work. Accessible tools that lower the technical barrier for deploying such designs are directly relevant to the infrastructure needs of digital health and mobile health research.

#### Technical Challenges for Researchers

Despite the promise of smartphone EMA, a persistent challenge is the technical burden it places on researchers who lack software engineering expertise. Building or customizing an EMA app requires cross-platform development skills, compliance with OS-specific notification systems, adherence to privacy regulations (eg, General Data Protection Regulation [GDPR]), and ongoing maintenance as operating systems update. These demands create barriers that delay study deployment and increase costs [14]. These obstacles can also lead to selection bias if the platform does not support certain device types or requires participants to have high digital literacy. Existing web-based and hybrid EMA solutions partially address this by offering browser-accessible interfaces or SMS-based prompting [15], but they often trade off rich sensor access, flexible notification logic, or the ability to support novel interaction modalities, such as gestures, against ease of setup [16].

#### Use of Gestures for Survey Responding

Phone gestures such as swiping to navigate or pinching to zoom have become a central part of modern smartphone user interface, where they replace many on-screen buttons and allow users to manipulate content through finger movements. Anecdotal evidence from UX research suggests that gesture-based interfaces feel intuitive and efficient, and that swiping in particular can increase engagement and enjoyment compared with purely button-based input [17,18]. However, experimental work shows that performance differences between taps and swipes are nuanced: some studies find comparable reaction times across gestures (Negulescu et al [19]), while others report longer reaction times for swipes than taps [20,21]. Swiping is at times favored over tapping [22], but this seems to depend on users’ previous smartphone experience, with more experienced users showing a stronger preference for swiping [23]. Within this broader landscape, our work treats swipe- versus tap-based answering as concrete examples of mobile gestures for survey responding, using them both as alternative interaction patterns and as a way to probe how well the SMAAT app and platform support fluent, gesture-based EMA surveys.

### Existing EMA Platforms and Gaps

In this section, we focus on a set of widely used, research-oriented EMA platforms that are representative of the broader landscape of smartphone-based EMA tools reviewed in recent overview papers [16,24]. To keep the comparison focused, we included here only general-purpose EMA platforms that (1) provide a web-based dashboard for researchers where they can configure a study without requiring custom programming and (2) deploy a dedicated smartphone app for participants. In addition, our previous work introduced Samply (developed by YS), an open-source web application that focuses specifically on scheduling and sending mobile notifications that redirect participants to externally hosted online surveys or experiments, thereby lowering the technical barrier for experience-sampling studies without providing a full in-app questionnaire engine [25].

Tables 1 and 2 summarize the selected EMA platforms along 2 groups of dimensions. Table 1 covers survey interaction capabilities – supported question types and notification options. Table 2 covers infrastructure features – passive sensing support, researcher dashboard and monitoring capabilities, and security features. In brief, LifeData (LifeData Corp), ExpiWell (Expimetrics Inc), and MetricWire (MetricWire Inc) focus on flexible time-based EMA scheduling with standard question formats (multiple-choice items, Likert scales, open text, and media), whereas movisensXS (movisens GmbH) offers particularly powerful sensor-triggered prompting in combination with Android-based questionnaires, and m-Path (m-Path Software bv) emphasizes EMA plus ecological momentary interventions in blended care settings [26]. JTrack-EMA+ (Research Centre Jülich) illustrates an open-source approach in which institutions host their own infrastructure and integrate EMA with broader smartphone-sensing pipelines [14]. Across these tools, data are typically protected via HTTPS and server-side encryption in vendor-managed or institution-managed clouds, but public documentation rarely describes full end-to-end encryption in which only the researcher controls the decryption keys.

**Table 1.** Question types and notification options of selected EMA^a^ platforms. Baseline shared by all platforms—question types: multiple-choice, rating scales, open-text responses, media with possible conditional branching and media upload from participants; notifications: interval-, signal-, and event-contingent schedules with push notifications. This table lists only what each platform adds or does differently.

Platform	Additional or distinctive question types	Additional or distinctive notifications supported
JTrack-EMA+	—^b^	+^c^ Reminders
m‑Path	+ Multiuser designs where participants can see or react to each other’s answers + Cognitive tasks	+ Reminders + Burst designs and automatic follow-up surveys + Geofencing-triggered notifications (via m-Path Sense)
movisensXS	—	+ Sensor-triggered notifications (wearable or device data) + Geofencing-triggered notifications
ExpiWell	+ Links to external web surveys or resources	+ Reminders + Geofencing-triggered notifications + SMS, email notifications
LifeData	—	+ Reminders
MetricWire	+ Cognitive tasks	+ SMS, email notifications, automated phone calls + Geofencing-triggered notifications
SMAAT^d^	+ Cognitive tasks + Swipe and tap responses + AI chatbots	+ Reminders + Geofencing-triggered notifications + SMS, email notifications

a EMA: ecological momentary assessment.

b No capabilities added on top of the shared baseline.

c “+” marks capabilities added on top of the shared baseline.

d SMAAT: sensor-based mobile app for assessment and tracking.

**Table 2.** Passive sensing, monitoring, and security features of selected EMA^a^ platforms. Baseline shared by all platforms—web dashboard: study or questionnaire setup, scheduling and sampling, participant management, compliance and response-count monitoring, CSV or data export; encryption: encrypted in transit and at rest. Passive sensing is rated minimal, limited, or extensive. This table lists only what each platform adds or does differently.

Platform	Passive sensing support	Web dashboard or monitoring	Security, encryption, or data privacy
JTrack-EMA+	Extensive—when used with the broader JTrack suite (eg, JTrack Social)	—^b^	GDPR^c^- and FAIR^d^-compliant, open-source (security depends on institutional deployment)
m-Path	Limited—sensing modules collect the data during a survey (for full sensing m-Path Sense app should be used)	+^e^ Gamification	GDPR- and HIPAA^f^-compliant
movisensXS	Extensive—integration with movisens wearables and environmental sensors	+ Graphical web editor for designing questionnaires and schedules	GDPR-compliant
ExpiWell	Limited—continuous GPS tracking, wearable and device usage integration	+ Built-in participant payments	GDPR- and HIPAA-compliant
LifeData	Minimal—some device or wearable integration in specific setups	—	GDPR- and HIPAA-compliant
MetricWire	Extensive—smartphone sensors, background location, integration with smartwatches or wearables	—	HIPAA- and PHIPA^g^-compliant
SMAAT^h^	Limited—smartphone sensors during the survey, background location, and health data	+ Built-in participant payments + Gamification	GDPR-compliant + end-to-end encryption

a EMA: ecological momentary assessment.

b No capabilities added on top of the shared baseline.

c GDPR: General Data Protection Regulation.

d FAIR: data are Findable, Accessible, Interoperable, and Reusable.

e “+” marks capabilities added on top of the shared baseline.

f HIPAA: Health Insurance Portability and Accountability Act.

g PHIPA: Personal Health Information Protection Act.

h SMAAT: sensor-based mobile app for assessment and tracking.

SMAAT is designed to address several converging gaps identified in this comparison. First, it provides a web-based study builder that supports not only standard item types but also advanced interactions, such as swipe- and tap-based responses and AI chatbots, which are not found in the other platforms. Second, SMAAT integrates fixed, random, interval, event-based, and geofencing notifications within a single no-code configuration interface, making complex trigger logic accessible without app development. Third, in addition to dashboards for compliance monitoring, response counts, question-level summaries, and sensor exploration, SMAAT implements an optional private key mechanism in which study data are encrypted with a researcher-controlled key so that the platform operator cannot decrypt stored datasets, approximating an end-to-end security model for research data at rest. This work evaluates the SMAAT platform in a cross-sectional usability study and lays the groundwork for longitudinal EMA deployments that leverage both innovative interaction modalities and complex notification logic.

### Objectives

This work has two main objectives. First, we aim to introduce SMAAT as a researcher-friendly EMA platform that integrates a web-based study designer, rich mobile question types, flexible notification logic, and secure data management for iOS and Android devices. Second, we report cross-sectional usability and user-experience findings from a study in which participants completed binary smartphone tasks using a swiping response format alongside traditional tapping, providing initial evidence on the feasibility and acceptability of swipe-based survey interactions in different populations.

## Methods

### Platform Description: SMAAT

#### Overall Architecture

SMAAT consists of a web-based platform for researchers [27] and a companion mobile app (SMAAT Research, developed by YS) for study participants. The researcher-facing component is a Next.js–based React application running on a Node.js server that provides the user interface for creating and managing studies, designing surveys, configuring notification schedules, and monitoring data collection. All study configurations and collected data are stored in a PostgreSQL database that is accessed via a standalone Keystone content management system, which serves as the backend for the platform. Communication between the frontend and backend is implemented using Apollo Client, which handles GraphQL queries and mutations between the Next.js application and the Keystone server.

The participant-facing mobile app is implemented in React Native using the Expo framework and is available for both iOS and Android devices for free as the “SMAAT Research” app in the respective app stores. When a participant enrolls in a study, the app retrieves the corresponding study configuration from the server, including study description, consent form, and any sensor settings that apply to that study. The app then delivers survey prompts to the participant according to the configured schedule and, when enabled, records sensor data during survey completion or in the background. Survey responses and sensor data are transmitted to the server once the smartphone is online, where they are stored in the central PostgreSQL database and made available through the researcher dashboard for visualization, export, and further analysis.

**Figure 1.**
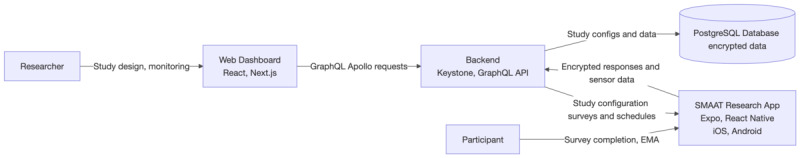
Overall architecture of the sensor-based mobile app for assessment and tracking (SMAAT) platform. High-level architecture of the SMAAT platform, showing the main components and their relationships.

From a high-level perspective, SMAAT’s architecture follows a client-server model with 3 main components: (1) the web dashboard used by researchers to define and monitor studies, (2) the mobile app used by participants to receive notifications and complete surveys, and (3) the backend services and database that coordinate configuration delivery, data ingestion, and storage (Figure 1). This modular design allows the same backend to support multiple concurrent studies and participant cohorts while keeping study configuration logic on the server and minimizing the need for custom app development for individual projects.

Unless otherwise noted, features described in this section are implemented and available in the current release of SMAAT but have not all been empirically evaluated in the studies reported in this paper; the cross-sectional study focused on survey delivery, usability, and swipe-based interaction. Evaluation of passive sensing, geofencing, AI chatbot, and end-to-end encryption features in operational EMA studies is planned as part of future work described in the Discussion section.

#### Study and Survey Builder

**Figure 2.**
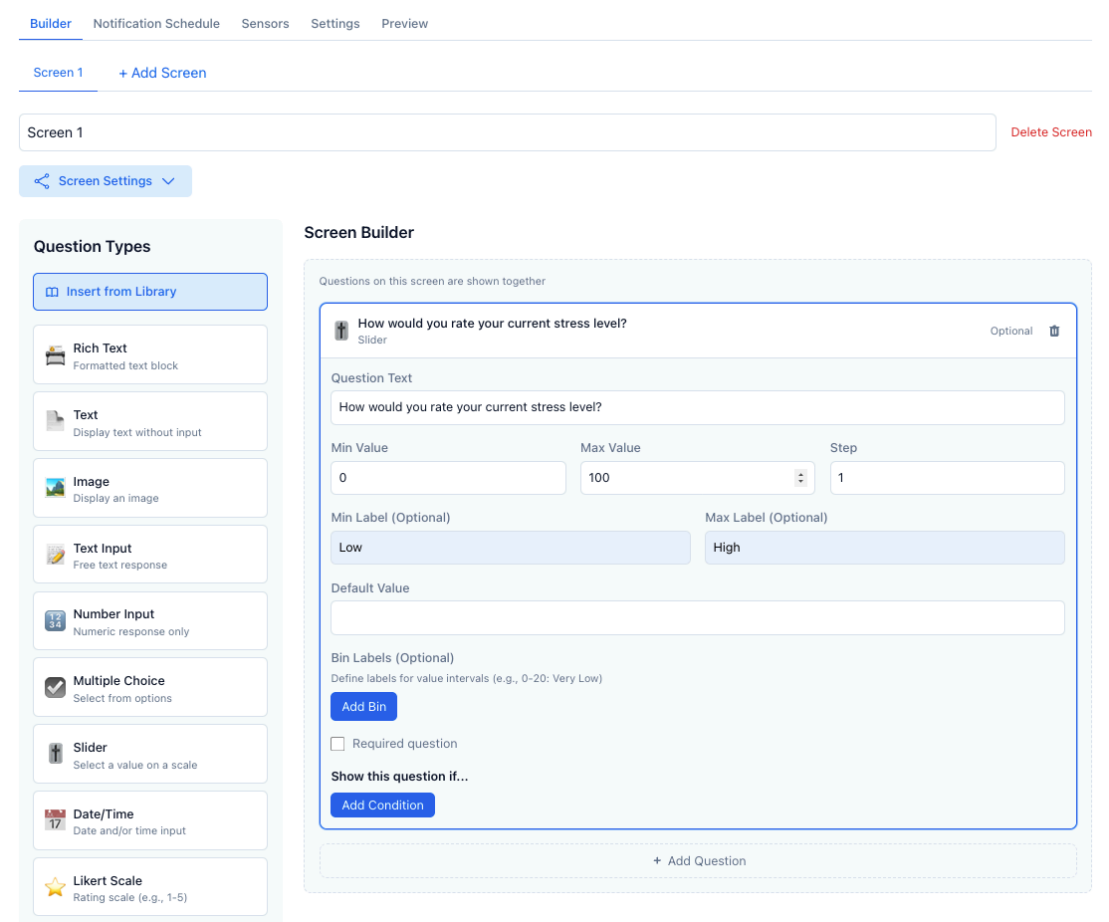
Screenshot of the survey builder.

Researchers configure studies in SMAAT through a visual web-based study and survey builder (Figure 2). Each study can be created from scratch or from a reusable template and includes basic metadata (eg, title, description, contact information, and study image), recruitment parameters, and the surveys and notification schedules associated with the study. The survey builder organizes surveys into screens, which allows researchers to structure multipage questionnaires with forward and backward navigation and to reuse or adapt existing configurations across studies.

The survey editor supports a range of standard item formats commonly used in psychological research, including static text or image blocks, open text and numeric input fields, multiple-choice questions, sliders, date and time inputs, Likert-type rating scales, sorting tasks, and file upload items. In addition, SMAAT includes mobile-friendly formats, such as swipe-based and tap-based binary decision items. Items can be added, removed, and reordered using drag-and-drop controls.

Among these formats, SMAAT includes a conversational item type (AI-based chatbot) based on a large language model. Rather than presenting a fixed question, this item conducts a short, bounded natural-language exchange with the participant and then returns structured data. At design time, the researcher specifies an instruction defining the chatbot’s role, one or more extraction goals (each with a target variable, data type, and guidance), and operational parameters such as the model type, the maximum number of conversational turns, and the response temperature. During the survey, the participant converses with the chatbot, which is steered by these goals to elicit the required information. On completion, the response is stored either as the verbatim transcript or, in a structured mode, as a JSON object produced by an additional deterministic extraction step that maps the conversation onto the researcher’s predeclared variables, so that conversational items yield data of the same form as conventional items. All model calls are proxied through the backend—the participant device never contacts the model provider directly nor holds its credentials—and per-participant rate limits and a per-study usage budget bound cost and burden.

The survey builder implements basic conditional logic and randomization features that are frequently required in experience sampling and experimental designs. Researchers can define simple branching rules so that specific questions or blocks are shown only if participants select particular response options or provide certain answers, and they can randomize the order of questions or randomly sample only a subset of questions on a given screen. The randomization of response options also helps mitigate presentation biases such as order and position effects; when enabled, the order of response options—in multiple-choice items and in the swipe and tap binary formats—is shuffled independently for each participant. The slider is treated specially to avoid presenting a starting anchor; if no default value is defined in the settings, the thumb is hidden until first interaction, the track is shown in a neutral color, and no value is displayed, so the participant sees an apparently empty scale rather than a suggestive starting point. Through these mechanisms — option-order randomization and suppression of default values — researchers can systematically limit the influence of biases such as anchoring, primacy, and position effects on the collected responses.

Importantly, the question-type system in SMAAT is modular. Each response format (eg, multiple choice, sliders, swipe or tap decisions, and AI chatbot) is implemented as a separate component with a shared interface, which allows the platform operator to add new response formats in the future with minimal changes to the overall architecture. Before starting a study, the survey builder provides a preview mode that demonstrates how questions and sensor prompts will be presented on mobile devices, allowing researchers to check navigation, layout, and item behavior without first enrolling as participants on a smartphone; this preview is not an exact reproduction of the final app design, so researchers are still encouraged to test the survey on real devices to evaluate the actual user experience.

#### Notification and Scheduling Logic

SMAAT provides a flexible push notification and scheduling system that supports several common patterns used in EMA and longitudinal mobile studies. For each survey, researchers can configure 1 or more notification schedules that determine when prompts are delivered to participants, how long a survey remains available after a notification is sent, and over which period of a participant’s study participation the schedule is active. Schedules can be limited to a defined duration that is specified relative to each participant’s enrollment time (eg, active for 14 d after enrollment), allowing individualized study periods without manual per-participant configuration.

The platform currently implements 5 main notification types: (1) fixed-time notifications sent at specific clock times, (2) random sampling within time windows, (3) interval-based schedules that repeat at regular time intervals with configurable maximum numbers of prompts per day, (4) event-based triggers that can be linked to specific events such as enrollment or study completion, to external events via API calls, or to predefined events within other surveys (eg, when a particular response option is selected), and (5) geolocation-based triggers that send a notification when participants enter or exit predefined geographic areas. For each notification type, researchers can specify availability windows during which the survey remains open after a prompt (eg, 30 or 60 min) and define reminders for unanswered notifications, which helps increase response rate.

Notification schedules are not evaluated on the fly but are materialized in advance as concrete, per-participant prompts, generated when a study is activated and when each participant enrolls. As a consequence, the sampling design can be adjusted while a study is running, and the platform gives the researcher explicit control over how such changes are applied to participants who are already enrolled: when a schedule is edited mid-study, the researcher can choose to reschedule all not-yet-delivered prompts under the new design, to add the new schedule while retaining the existing prompts, or to apply the change only to participants enrolled thereafter. Prompts that have already been delivered are never altered by these operations. This allows researchers to make mid-course corrections—such as adjusting prompt frequency or availability windows—while deciding deliberately whether the change should affect the ongoing experience of currently enrolled participants or only that of new enrollees.

In the cross-sectional study reported in this paper, we did not use push notifications; instead, participants accessed the survey directly from the main screen of the SMAAT Research app after installing it and providing consent. Future EMA deployments using SMAAT can combine these notification types (eg, random daytime prompts, fixed evening prompts, and geofenced triggers) within a single study.

#### Data Collection and Security

In SMAAT, data collection can involve both active survey responses and, when enabled, passive sensor measurements recorded during or between surveys. When a participant completes a survey on the SMAAT Research app, their responses and any associated sensor readings configured for that study are stored locally on the device until the internet connection allows them to be transmitted to the server. This design allows SMAAT to operate offline; because each study’s configuration is cached on the device after enrollment, participants can open and complete questionnaires and record the associated sensor data without network connectivity, and the completed responses are held in an encrypted on-device queue and synchronized automatically once a connection becomes available, so that intermittent connectivity does not lead to data loss. Data transfer between the mobile app, the web dashboard, and the backend services uses HTTPS with Transport Layer Security to protect data in transit.

To preserve the temporal integrity of the data, each response is associated with timestamps from 2 independent clocks. The device records a local “time of event”—the start and completion times of the survey and a timestamp for each individual response—generated from the smartphone’s own clock, together with the device’s timezone. In addition, at the moment a response is received by the backend, the server records an independent, trusted “time of arrival” from the server clock, and both timestamps are stored together on every record. Retaining the device time preserves the participant’s local event time, which is important when responses are collected offline and synchronized later, while the server timestamp provides a tamper-resistant reference that is unaffected by an incorrect or manipulated device clock. For time-sensitive decisions, such as compliance (that is, whether a response falls within a notification’s availability window), SMAAT relies on the server clock rather than the device clock. Compliance is based on the interval between the server-recorded notification dispatch time and the server time of arrival, ensuring that an erroneous local time cannot affect compliance classification, and the 2 timestamps can additionally be compared during analysis to identify responses with implausible clock skew.

To keep collected data interpretable when a study’s design is modified during data collection, each stored response also retains a snapshot of the item as it was presented—including the question text and type at the moment of answering and the order in which screens and questions were actually shown to that participant (reflecting any randomization or conditional branching). Editing or reorganizing a survey after enrollment therefore does not retroactively alter or orphan responses collected beforehand, since the original wording and presentation order travel with each response.

Sensor data are acquired through cross-platform interfaces (the Expo sensor and location APIs) that wrap the native iOS and Android sensing frameworks and return each sensor in a standardized unit and schema, so that accelerometer, gyroscope, magnetometer, barometer, and location data from both operating systems are stored in a directly comparable form. The sampling configuration for each sensor (update interval and, for GPS, the requested accuracy and distance thresholds) is defined by the researcher at the study level and applied identically across devices. SMAAT stores the full-resolution raw sensor streams together with detailed device metadata—operating system and version, manufacturer, model, and a device performance class—recorded at enrollment and refreshed with each response, and does not apply irreversible on-device transformations to the raw signal. This design harmonizes interface-level differences between platforms while preserving the original data, so that researchers can apply study-specific normalization or calibration (eg, standardizing inertial-sensor axes, applying model-specific offsets, or filtering location fixes by reported accuracy) during analysis using the accompanying device metadata.

For the conversational item type (AI-based chatbot), generating the chatbot’s replies and performing structured extraction requires transmitting the conversation to an external language-model provider acting as a subprocessor; this content is encrypted in transit and stored under the same protections as other responses, but is processed in readable form by the provider. The conversational item is opt-in and disabled by default; no data is transmitted to the external provider unless the researcher both configures a provider key for the study and includes such an item in a survey, and because each study uses the researcher’s own provider account, the provider-side handling of the data is governed by the researcher’s agreement with that provider. The platform offers optional safeguards for this processing—a configurable AI-specific consent step, best-effort redaction of common identifiers before transmission, and optional content moderation with crisis-resource surfacing—but researchers using conversational items remain responsible for disclosing third-party AI processing in their consent materials, establishing the appropriate data-processing agreement, and designing such items so as not to elicit unnecessary identifying information.

On the server side, incoming data are written to a PostgreSQL database in which sensitive fields are encrypted at rest, and database access is restricted to the backend services that handle authentication, study configuration, and data export. Researchers access collected data through the web dashboard and do not have direct access to the underlying database, which separates study management from low-level data storage. On top of this baseline, SMAAT lets researchers choose, per study, how participant data are encrypted. Under server-side encryption (intended for piloting and platform testing), data are encrypted in transit and at rest under keys managed by the platform, which can therefore technically read the content while acting as the researcher’s data processor. Under end-to-end encryption—the recommended default—data are encrypted on the participant’s device before transmission: each dataset’s content key (AES-256-GCM) is wrapped, using X25519/ECIES, to a per-study key that only the researcher’s account can open, so the platform stores only ciphertext and cannot read the study’s content. This key is unlocked automatically when the researcher signs in—there are no key files to manage—and is protected by the researcher’s account password with a 1-time recovery code as the only backup; the platform never receives the password or a usable copy of the private key, and authorized collaborators can be granted decryption access without exchanging key files. For researchers who prefer to hold their own keys, SMAAT also supports a self-managed study key: the platform generates a public-private key pair, shares the public key with participant devices to encrypt collected data, and shows the private key to the researcher only once without storing it; securely retaining this key is then the researcher’s responsibility, and if it is lost the encrypted data cannot be decrypted. In both end-to-end modes, the platform cannot recover a lost key or recovery code, so these options are chosen by researchers who require this level of protection and understand its implications; metadata needed to operate the service (such as timestamps and study or account identifiers) remain readable regardless of the option selected.

The overall design follows general data protection principles (eg, secure communication channels, encryption at rest, and optional end-to-end encryption) and is intended to support studies conducted under the European Union GDPR and similar institutional and regional data protection requirements.

#### Researcher Dashboard and Visualizations

The web-based researcher dashboard serves as the central interface for managing studies and inspecting incoming data in SMAAT. After logging in, researchers can view an overview of all ongoing and completed studies, create new studies, and access study-specific pages for survey configuration, participant management, and data monitoring. The study dashboard summarizes key operational metrics such as the number of invitations sent, active participants, notification counts, and completed surveys, helping researchers track recruitment and field progress in real time.

For data inspection and analysis, SMAAT provides several built-in data views. A data overview interface offers high-level summaries of study data, including counts of completed surveys, temporal patterns of responses, and basic distributions of selected variables. Researchers can create configurable data dashboards with visualizations, such as line charts and pie charts, to compare participant engagement across time or across studies, and they can generate data reports for export and further analysis in external statistical software. In addition, study-level views include compliance and response-rate visualizations that highlight, for example, the proportion of delivered prompts that were answered per day or per participant, which supports early detection of adherence problems during EMA studies. More advanced analytics and visualization features are under active development but are beyond the scope of the current manuscript.

### Empirical Evaluation: Cross-Sectional Study

#### Study Design and Procedures

We conducted a cross-sectional, between-participants study to evaluate a swiping response format for binary survey questions implemented in the SMAAT Research app, using a conventional tapping format as a comparison condition. The study was run in 2 independent samples: first-year psychology students at the University of Konstanz who participated for course credit and online panel participants from Prolific in the United States who participated for monetary compensation. Participants in each sample were randomly assigned to one of two response formats (swipe vs tap) and to one of two response orientations (yes on the left vs yes on the right), resulting in a 2×2 between-participants design. After installing the app, participants completed the study in a single session on their own mobile devices (smartphones or tablets); no push notifications were used, and the survey was accessed directly from the main screen after consent.

The survey design was identical for both samples. First, participants were presented with study information and an informed consent form within the app. Next, they completed demographic and smartphone-related questions (eg, age, gender, years of owning a smartphone, and familiarity with smartphones). Participants then answered several blocks of binary questions using their assigned response format and orientation: personality items from a brief version of the Eysenck Personality Inventory, climate change facts, climate change attitude statements, and parity judgments (odd-even number classification), and in the sample of Prolific users, an additional block of climate action tips. After completing the binary question blocks, participants filled out usability items based on the System Usability Scale (SUS) and user-experience items from the short version of the User Experience Questionnaire (UEQ-S), along with open-ended questions about their overall survey experience. Upon finishing the survey, participants were debriefed and received course credit or monetary compensation according to the recruitment source.

#### Participants

##### Sample 1: University Students

Sample 1 consisted of 97 first-year psychology students at the University of Konstanz in Germany who completed the study as part of a course activity. Participants were between 18 and 31 years old (mean 20, SD 2 y), and the sample included 83 women, 10 men, 2 participants who identified as diverse, and 2 participants who did not report their gender. Students were informed that their participation in this specific study was voluntary within the course context. Inclusion criteria were being enrolled as a first-year psychology student at the university, owning a mobile device (smartphone or tablet) compatible with the SMAAT Research app, and providing informed consent.

##### Sample 2: Prolific Participants

Sample 2 consisted of 132 participants recruited via the Prolific online platform and residing in the United States. Participants were between 20 and 75 years old (mean 41, SD 12 y), and the sample included 66 women, 63 men, 2 participants who identified as diverse, and 1 participant who preferred not to answer. Prolific participants were compensated with monetary payments in line with platform and ethical guidelines. Eligibility criteria included being an adult Prolific user based in the United States, owning a compatible mobile device (smartphone or tablet), and providing informed consent within the SMAAT Research app.

### Measures

We recorded response-time and accuracy measures for each binary item block to assess performance differences between the swiping and tapping formats. Response time was defined as the latency between item presentation and the registered response, and accuracy was computed for blocks with objectively correct answers (climate change facts and parity judgments).

Usability was assessed with items based on the SUS [28], using 5-point Likert-type response options to rate agreement with statements about the ease of use of the assigned response format. User experience was measured with the UEQ-S [29], which provides pragmatic and hedonic quality scores on 7-point semantic differential scales. At the end of the survey, participants also answered open-ended questions about their overall impression of the survey experience and the swiping or tapping format, which were later inspected qualitatively to identify recurring themes.

### Ethical Considerations

The study was reviewed and approved by the Institutional Review Board (Ethics Committee) of the University of Konstanz (IRB statement 39/2025). The committee evaluated the project “Swipe to Answer: Evaluating Swiping as an Innovative Method for Binary Survey Data Collection on Smartphones” and concluded that the procedures for data handling and storage were in full accordance with the ethics regulations of the University of Konstanz, the Declaration of Helsinki in its current version, and relevant national and international law and regulations. The approval was granted for a period of 5 years, including additions and prolongations of a technical nature that do not affect the overall ethical assessment or the safety of study participants.

All participants were informed about the study objectives, procedures, and data protection measures within the SMAAT Research app and provided electronic informed consent before any data were collected. Participation was voluntary, and participants could discontinue at any time without penalty or loss of benefits. Data were collected and stored in line with the institutional procedures and data protection framework reviewed by the ethics committee.

### Statistical Analysis

Analyses were conducted for the student and Prolific samples. For each participant and task block, we computed mean response times and accuracy proportions. Response-time outliers were removed by excluding values more than 2 SDs above or below the mean for the respective participant. To examine performance differences between response formats, we compared swiping and tapping conditions on response-time and accuracy measures using analysis of variance with response format and response orientation as fixed factors.

Usability and user-experience scores were analyzed by computing total SUS scores and pragmatic and hedonic UEQ-S scores for each participant and comparing these across response formats and response orientations with similar between-participants analyses. Where appropriate, we report effect sizes and CIs, and we focus interpretation on the pattern and magnitude of effects rather than on statistical significance alone. Open-ended comments were inspected qualitatively to identify recurring themes in participants’ experiences with the swiping and tapping formats, but no formal qualitative coding framework was applied.

## Results

### Platform Feasibility and Study Execution

#### Study Setup in SMAAT

The study for both samples (students and Prolific participants) was fully configured and executed using the SMAAT web dashboard, including creation of study entries, survey blocks, and response formats (tapping vs swiping) for participants. We used the survey builder to implement the surveys (personality, climate change facts and attitudes, parity judgments, and climate action tips) and pretested the mobile layout before conducting the study. Study configurations were delivered successfully to the participant app on both iOS and Android devices, and the app handled enrollment, consent, survey access, and data synchronization without major technical failures reported by participants or observed in the logs.

#### Recruitment and Completion

In both samples, participants joined the study via a SMAAT study web page that displayed a QR code and a study code; they could either scan the QR code with the app or manually enter the study code to enroll. In sample 1, first-year university students were invited during an introductory psychology class and completed the study on their own smartphones in the classroom. In sample 2, participants were recruited via Prolific and accessed the same type of SMAAT study web page through a study link on the platform. Across both samples, all enrolled participants completed the full survey protocol in a single session, indicating that end-to-end recruitment, app installation, and survey completion were feasible within the SMAAT workflow. Completion time stamps and server logs showed that data transfers from the mobile app to the web platform proceeded as expected, and we could monitor participation and completion status in real time via the researcher dashboard.

### Sample Characteristics

#### Demographics and Smartphone Use

Across both samples, we analyzed data from university students (sample 1) and Prolific participants (sample 2), with each participant contributing multiple trial-level responses but only one set of background and usability ratings. In sample 1, students were typically in their early twenties, with ages ranging roughly from late adolescence to the early thirties (mean 20.2, SD 2.05, range 18‐31 y). In sample 2, Prolific participants covered a broader adult age range from young to older adulthood (mean 41.4, SD 12.4, range 20‐75 y). Within each sample, gender distribution was skewed toward women in the student sample (n=83, 85.6%) and more balanced in the Prolific sample (n=66, 50%), reflecting typical patterns in psychology participant pools and online panels. Most participants in both samples reported owning a smartphone for several years (often “more than 5 years,” n=84, 86.6% in student sample and n=124, 93.9% in Prolific sample) and rated their familiarity with smartphones in the upper part of the response scale, indicating that they were comfortable using mobile apps (student sample: mean 4.04, SD 0.63, Prolific sample: mean 4.85, SD 0.41). Together, these characteristics suggest that both samples were well-experienced for evaluating the SMAAT smartphone survey workflow.

#### Device and Context

There were more iOS than Android users in both samples; student sample: Android=31 (32%), iOS=66 (68%), Prolific sample: Android=51 (39%), iOS=81 (61%). In the student sample, 70 participants (72%) used smartphones and 27 participants (28%) used tablets. In the Prolific sample, all participants used smartphones. SMAAT handled this heterogeneity without technical issues.

### Task Performance

Across both samples, response patterns indicated that the binary tasks were performed as intended when delivered via SMAAT. Accuracy on the objective tasks—climate change facts and parity judgments—was generally high in both samples (student sample: 873/963, 91% correct answers on climate change facts and 836/837, 99.9% correct answers in parity judgments; Prolific sample: 1112/1310, 85% correct answers on climate change facts and 1178/1179, 99.9% correct answers in parity judgments), suggesting that participants understood the items and could respond reliably on their smartphones. A 3-way ANOVA on proportion correct with sample (students vs Prolific), response format (tap vs swipe), and response orientation (yes left vs yes right) as between-participants factors revealed no significant main effects of sample (*F*_1,221_=1.31; *P*=.25; *η*_p_^2^=0.04, 95% CI 0.00-0.10), response format (*F*_1,221_=0.01; *P*=.90; *η*_p_^2^<0.01, 95% CI 0.00-0.10), or response orientation (*F*_1,221_=0.01; *P*=.94; *η*_p_^2^<0.01, 95% CI 0.00-0.04). None of the 2-way or 3-way interactions reached significance (all *F*<0.43, all *P*>.51), with very small effect sizes (*η*_p_^2^≤0.01), indicating that accuracy did not depend on the combination of sample, response format, or response orientation. For example, accuracy ranged from mean 0.91 (SD 0.08) for Prolific participants using tapping with “yes” on the right to mean 0.95 (SD 0.05-0.07) for students across all response formats and orientations.

When comparing response formats in terms of the response time, swiping and tapping yielded broadly comparable performance, with no evidence of systematic disadvantages of swiping for either accuracy or overall task completion. A three-way ANOVA on participants’ mean response times with sample (students vs Prolific), response format (tap vs swipe), and response orientation (yes left vs yes right) as between-participants factors revealed no significant main effect of sample (*F*_1,221_=1.91; *P*=.17; *η*_p_^2^=0.01, 95% CI 0.00-0.05), and no significant main effect of response format (*F*_1,221_=3.53; *P*=.06; *η*_p_^2^=0.01, 95% CI 0.00-0.06). The main effect of response orientation reached conventional significance (*F*_1,221_=4.23; *P*=.04; *η*_p_^2^=0.01, 95% CI 0.00-0.05), but the corresponding Tukey-adjusted contrast between left and right “yes” positions was not significant (mean difference=−477 ms, 95% CI −1035 to 82; *t*_221_=−1.69; *P*=.09), and the effect size was small. None of the 2-way or 3-way interactions involving sample, response format, and orientation were significant (all *F*<3.75, all *P*>.05, with *η*_p_^2^≤0.01), suggesting that any orientation-related differences were weak and unstable and that swiping and tapping yielded broadly comparable response times across samples. Descriptively, response times were slower for students (eg, tap-left mean 4189 ms, SD 4757 ms; tap-right mean 5388 ms, SD 20035 ms; swipe-left mean 5471 ms, SD 7187 ms; swipe-right mean 6172 ms, SD 9347 ms) than for Prolific participants (tap-left mean 3369 ms, SD 8580 ms; tap-right mean 3069 ms, SD 4743 ms; swipe-left mean 3136 ms, SD 4370 ms; swipe-right mean 3445 ms, SD 9756 ms), but these differences were not statistically reliable in the factorial model.

Taken together, these results indicate that SMAAT can deliver survey tasks with adequate data quality using both tapping and swiping, providing a sound basis for further applications in longitudinal designs.

### Usability and User Experience

**Figure 3.**
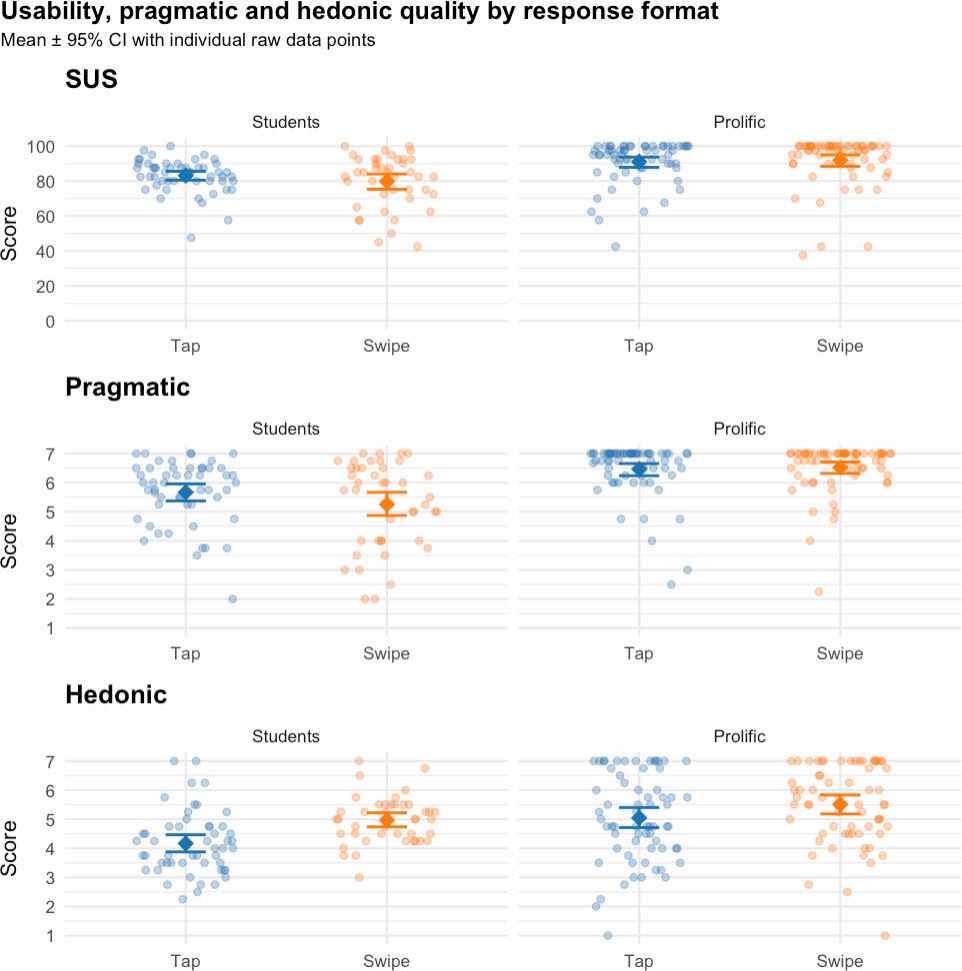
Usability and user experience scores.

We analyzed overall usability (SUS total score) and pragmatic and hedonic user experience (UEQ-S means) using 3-way ANOVAs with sample (students vs Prolific), response format (tapping vs swiping), and response orientation (yes on the left vs yes on the right) as between-participants factors (Figure 3).

SUS scores were in the acceptable range across all conditions, with students showing means around 80‐83 and Prolific participants around 91‐92. We analyzed SUS usability scores with a 3-way ANOVA including sample (students vs Prolific), response format (tap vs swipe), and response orientation (yes left vs yes right) as between-participants factors. The main effect of sample was significant (*F*_1,210_=4.80; *P*=.03; *η*_p_^2^=0.02, 95% CI 0.00-0.08), indicating that Prolific participants rated the app as more usable than students. In contrast, the main effects of response format (*F*_1,210_=0.20; *P*=.65; *η*_p_^2^<0.01, 95% CI 0.00-0.01), and response orientation (*F*_1,210_=0.01; *P*=.99; *η*_p_^2^<0.01, 95% CI 0.00-0.02), were not significant, and all interactions were nonsignificant (all *F*<0.30, all *P*>.59), with very small effect sizes. Estimated marginal means showed that students reported an average SUS score of 81.6 (SE 1.42, 95% CI 78.8-84.4), whereas Prolific participants reported 91.5 (SE 1.16, 95% CI 89.28-93.8), with a mean difference of −9.92 (95% CI −13.51 to −6.33; *t*_210_=−5.41; *P*<.001). Descriptively, SUS scores were in the acceptable-to-good usability range across all conditions (students: mean 81.60, SD 12.40; Prolific: mean 91.50, SD 13.10), suggesting that neither swiping versus tapping nor “yes” orientation impaired perceived usability.

UEQ-S pragmatic quality scores were analyzed with a 3-way ANOVA including sample (students vs Prolific), response format (tap vs swipe), and response orientation (yes left vs yes right) as between-participants factors. The main effect of sample was significant (*F*_1,216_=8.46; *P*=.004; *η*_p_^2^=.04, 95% CI 0.01-0.10), indicating higher pragmatic quality ratings among Prolific participants than among students. Neither the main effect of response format (*F*_1,216_=0.06; *P*=.80; *η*_p_^2^<0.01, 95% CI 0.00-0.03), nor the main effect of response orientation (*F*_1,216_=0.52; *P*=.47; *η*_p_^2^<0.01, 95% CI 0.00-0.03), was significant, and all interactions were nonsignificant (all *F*<0.67, all *P*>.54), with very small effect sizes. Estimated marginal means showed that students reported a mean pragmatic score of 5.52 (SE 0.11, 95% CI 5.30-5.75), whereas Prolific participants reported 6.49 (SE 0.09, 95% CI 6.31-6.67), with a mean difference of −0.97 (95% CI −1.26 to −0.69; *t*_216_=−6.63; *P*<.001). Descriptively, pragmatic quality ratings were positive in all conditions (students: mean 5.48, SD 1.29; Prolific: mean 6.50, SD 0.88), with no suggestion that swiping versus tapping or “yes” orientation impaired perceived pragmatic quality.

UEQ-S hedonic quality scores were analyzed with the same 3-way ANOVA including sample (students vs Prolific), response format (tap vs swipe), and response orientation (yes left vs yes right) as between-participants factors. The main effect of sample approached but did not reach conventional significance (*F*_1,216_=3.36; *P*=.07; *η*_p_^2^=0.02, 95% CI 0.00-0.06), suggesting somewhat higher hedonic ratings among Prolific participants than students. The main effects of response format (*F*_1,216_=1.65; *P*=.20; *η*_p_^2^=0.06, 95% CI 0.01-0.13), and response orientation (*F*_1,216_<0.01; *P*=.98; *η*_p_^2^<0.01, 95% CI 0.00-0.03), were not significant, and all interactions were nonsignificant (all *F*<1.82, all *P*>.18), with small effect sizes. Descriptively, hedonic ratings tended to favor swiping over tapping in both samples (students: tapping mean 4.17, SD 1.09 vs swiping mean 4.97, SD 0.81; Prolific: tapping mean 5.05, SD 1.49 vs swiping mean 5.51, SD 1.33), but these differences were not statistically reliable in the factorial model.

### Qualitative User Feedback

Across both samples, many participants described the overall survey design as clear, easy, and quick to complete, regardless of whether they used tapping or swiping. Typical positive comments emphasized the simplicity and speed of the interface (eg, “It went quickly” and “Very easy to understand and use”), and several Prolific participants explicitly praised the interface as intuitive and more efficient than traditional multiple-choice layouts. At the same time, a recurring theme in both student and Prolific comments was that strict yes or no answers leave “too little room” or “no opportunity for nuance,” especially for personality and attitude questions, indicating a perceived limitation of the binary response format rather than the SMAAT implementation itself.

Feedback on the swiping response method was often explicitly positive, with students describing swiping as “easy and fun” or “enjoyable,” and Prolific participants calling it “fun,” “engaging,” and “interesting” while still “easy to answer.” Several participants, however, noted that swiping can lead to occasional mis-swipes or a desire for an “undo” option (eg, “I liked the swiping, but disliked that you could not go back after swiping”), and a few student respondents expressed concern that older people might be less familiar with swiping gestures. Comments about tapping were also largely positive, highlighting that tapping felt straightforward, fast, and accurate (eg, “It was easy to tap my answer and everything flowed nicely”), although some Prolific participants found it “boring and hardly innovative” or suggested small refinements such as clearer highlighting of selected answers or better cues to scroll when additional items were present on a page.

Taken together, the qualitative feedback aligns with the quantitative usability results in indicating that SMAAT’s binary interfaces are perceived as simple and efficient, with swiping adding a more enjoyable and “modern” interaction at the cost of occasional concerns about mis-swipes and a lack of nuanced response options.

Overall, the combination of high usability ratings, largely positive user feedback, and the absence of systematic performance disadvantages for swiping suggests that SMAAT is suitable for use in more demanding longitudinal EMA studies, where long-term engagement and efficient mobile interaction are critical.

## Discussion

### Principal Findings

Our findings show that SMAAT can reliably support smartphone-based surveys and that swiping is a viable alternative to tapping that offers usability and user experience comparable with traditional response formats. Across 2 independent samples and multiple task types, we observed acceptable SUS scores, high pragmatic user-experience ratings, and largely positive qualitative feedback for both formats, with descriptive advantages for swiping on hedonic user experience but no systematic performance disadvantages in terms of accuracy or overall task completion. Together with smooth end-to-end study execution via the SMAAT dashboard and mobile app, these results indicate that SMAAT and the swiping response format are well suited for future longitudinal EMA studies that require repeated, efficient, and engaging data collection on participants’ own devices.

Beyond these specific usability outcomes, this work illustrates SMAAT’s broader contribution as a flexible EMA platform that integrates rich question types, configurable notification logic, and secure data management. We demonstrated that complex survey tasks combining multiple binary blocks, usability scales, and open-ended questions can be configured through the web dashboard and deployed to heterogeneous devices in different populations (university students and a US Prolific sample) without major technical failures. The study also provides evidence that swipe-based response formats are usable and engaging when implemented within SMAAT, and at the same time documents the technical feasibility of managing multiblock smartphone surveys entirely through the researcher platform.

### Comparison With Previous Work

SMAAT builds on and extends existing EMA platforms such as JTrack-EMA+ and related tools by combining a broad palette of active question types with support for passive sensor data and advanced notification logic. In addition to conventional item formats (single- and multiple-choice, sliders, Likert scales, and text and file uploads), SMAAT supports swiping as a response method and includes an AI chatbot that can be used for surveying participants using conversational prompts, which is uncommon in current EMA solutions. On the prompting side, the platform offers fixed, random, interval, event-based, and geolocation-based notifications, allowing researchers to implement complex triggers without custom programming.

At the level of study monitoring and analysis, SMAAT provides integrated dashboards for visualizing compliance, response counts over time, and basic answer distributions, as well as exportable CSVs for more advanced modeling in external software. These features align with and extend previous work on EMA infrastructure that emphasizes the importance of flexible scheduling, real-time compliance tracking, and prompt reacting to noncompliance for high-quality longitudinal data [7]. Our usability findings also dovetail with previous research on mobile interaction modalities, which has generally found that gesture-based inputs on smartphones can deliver high usability and engagement [17,18]; here we add evidence that a swipe-based interface is comparable with a tap-based interface in pragmatic usability and tends to be experienced as more enjoyable, at least descriptively.

### Implications for EMA Research

From an EMA perspective, the results of this study suggest that SMAAT can lower several practical barriers to running cross-sectional or longitudinal smartphone studies. First, the combination of a visual web-based survey builder, multiple notification types, and built-in handling of consent, permissions, and secure data synchronization means that researchers can implement relatively complex EMA designs without building their own app or backend. Second, the high SUS scores, strong pragmatic UEQ-S ratings, and largely positive qualitative feedback for both tapping and swiping indicate that participants are comfortable interacting with SMAAT on their own devices, which is critical for sustained EMA participation.

The descriptive hedonic advantage of swiping, together with comments that described it as fun, modern, and engaging, points to swiping as a promising option for EMA protocols that may otherwise risk fatigue or disengagement over time. At the same time, participant feedback about occasional mis-swipes and the desire for an “undo” function offers concrete design guidance for improving future studies (eg, by adding an optional confirmation step or an easy way to correct the last response). Looking ahead, SMAAT’s support for passive sensors, geofencing, and event-based notifications creates opportunities to combine swipe-based binary responses with contextual triggers in domains such as mental health, behavior change, or daily decision-making; however, these applications should be treated as future directions to be tested in dedicated longitudinal studies rather than as claims supported by the current cross-sectional data.

Beyond its methodological contributions, SMAAT has direct relevance as a digital health research tool. EMA platforms that are accessible to research groups without app-development capacity can accelerate deployment of studies in clinical and community health settings (eg, studies of mood regulation, symptom monitoring, medication adherence, or health behavior change). SMAAT’s support for GDPR-compliant data handling, optional end-to-end encryption, and flexible notification schedules (including event-based and geofenced triggers) makes it suitable for health research contexts where data sensitivity and participant safety are primary concerns. The combination of validated usability and pragmatic user experience across heterogeneous devices and populations also suggests that participants with varying levels of digital literacy can engage with the platform effectively, which is an important consideration for real-world clinical and public health applications.

### Limitations

Several limitations of this work should be acknowledged. First, this study used a cross-sectional design in which participants completed a single session of survey tasks; we did not yet deploy true longitudinal EMA protocols or collect continuous sensor streams, so conclusions about long-term feasibility and data quality in EMA settings remain preliminary. Second, although the platform now records notification delivery, dismissal, and application-error outcomes, we did not systematically track or analyze these signals—or other technical metrics such as battery usage and background sensor missingness—in this first evaluation, which limits our ability to quantify the technical footprint of SMAAT in everyday use. Relatedly, although sensor readings are stored in standardized units with accompanying device metadata, we did not empirically benchmark the accuracy and comparability of sensor measurements across the heterogeneous iOS and Android hardware used by participants, and the platform does not currently apply hardware-level calibration to correct for such differences.

Third, the samples consisted of a convenience group of first-year university students and an online US panel from Prolific, which restricts the generalizability of our findings to other age groups, cultural contexts, and populations with lower smartphone literacy. Finally, while the current version of SMAAT includes core dashboards for monitoring study progress and exploring response distributions, more advanced analytics and visualization features are still under development, meaning that researchers presently need to export data for many types of in-depth statistical analyses.

### Future Work

The findings of this study are limited to single-session, cross-sectional data and therefore do not address how swipe-based responding performs when used repeatedly over longer periods. Work on microinteraction EMA using smartwatches has shown that very brief prompts that can be answered with a quick glance and a single tap can achieve high adherence and low perceived burden over several weeks, even at higher prompt frequencies than typical phone-based EMA. These studies suggest that extremely lightweight interactions can support long-term compliance, but they do not directly speak to repeated swiping in smartphone EMA. In our data, swiping was accurate, efficient, and well-received in a single session, yet it remains an open question whether, over days or weeks, swiping leads to different trajectories of response times, error rates, or adherence than conventional tapping. Addressing these longitudinal dynamics—how performance and compliance evolve over time for swiping versus tapping—is a goal for planned EMA studies using SMAAT.

In parallel, we plan dedicated methodological work on SMAAT’s passive sensing and technical performance, focusing on the quality and completeness of sensor data (eg, GPS), battery impact under different sampling regimes, and reliability of background operation and notification delivery. This work will include empirical benchmarking of sensor data across representative iOS and Android devices to characterize cross-device heterogeneity and to inform appropriate normalization procedures. Such validation is particularly relevant given that platform-specific differences between Android and iOS can materially affect mobile data collection, as we observed for background location handling in earlier work on geofencing with the Samply app [30].

As the analytics and visualization modules of the platform mature, future papers will document and validate advanced dashboard features for monitoring compliance, visualizing trajectories, and supporting exploratory and confirmatory analyses directly within SMAAT. Once the platform has been fully validated in these methodological studies, we envision domain-specific apps—for instance, in mental health, behavior change, or environmental attitudes—that leverage EMA designs and context-aware triggers, but these apps lie beyond the evidential scope of this work and should be developed in collaboration with domain experts.

### Potential Applications and Impact

The combination of flexible EMA settings and sensor connectivity in SMAAT makes it well-suited for applications that depend on frequent, real-time input from everyday users rather than trained experts. This approach fits well with the growing trend of mobile app–based citizen science. Earlier studies on citizen science platforms, especially in environmental and sustainability research, have shown that mobile tools can motivate people to share data often while also helping them feel more aware of and involved in local challenges [31].

Hognogi et al [32] highlighted the role of smartphones as monitoring tools and participatory interfaces and identified major fields where citizen science could be applied: (1) environmental and public health risk monitoring (eg, water quality, radiation, noise, waste, natural hazards, infectious diseases, and allergens), (2) biodiversity monitoring and nature connectedness (eg, species observations of birds, insects, plants, invasive species, and AI-based species identification), (3) well-being and urban environmental quality (eg, thermal comfort, urban heat islands, green spaces, mobility, smart cities, and cultural heritage management), (4) agriculture and primary or secondary sector resource management (eg, crop monitoring, forestry, fisheries, and mining-related monitoring), and (5) linguistic and cultural data collection (eg, recording free speech). Given that Hognogi et al [32] identified repeated field-based data collection as a core citizen science need, SMAAT’s EMA and geofencing features address that need directly in the majority of fields mentioned above.

Another potential application of SMAAT is in the domain of usability testing. SMAAT could support frequent, low-effort responses that complement sensor data. For example, participants in water filter usability testing could repeatedly report on perceived taste of water, its clarity, or odor when changing filters, or rate their satisfaction with drinking water alongside smart filter monitoring. Wen et al [33] highlighted the importance of study design and long-term compliance strategies in usability research. This underscores the value of user-centered, long-term engagement approaches—an area where SMAAT’s brief, repeated prompts in combination with gamification features could contribute meaningfully.

One of the strengths of SMAAT is its potential to track and nudge specific behaviors, making it a relevant tool for supporting behavioral change in sustainability domains. Kaufman et al [34] identified several barriers to behavior change in sustainability transitions, including the fact that many relevant behaviors are automatic and habitual rather than reflective; overcoming this barrier requires first making such routines visible and then disrupting them with targeted interventions. For example, SMAAT could provide interventions to prompt users to wash clothes at lower temperatures, separate waste, or choose more sustainable products. This aligns with Mosca et al [35], who identified digital tools such as power metering and gamification as effective means of promoting sustainable behavior. For future development, SMAAT’s potential ability to integrate with IoT sensors means it could complement these approaches and support long-term behavioral change in everyday contexts.

### Conclusions

SMAAT emerges from this work as a promising and flexible EMA platform that can deliver complex smartphone surveys with rich question types, configurable notification logic, and integrated data management entirely through a web-to-app workflow. Using a cross-sectional study with university students and a US Prolific sample, we showed that a swipe-based response format can be implemented within SMAAT alongside traditional tapping and that both formats achieve good usability, high pragmatic user-experience ratings, and largely positive participant feedback without systematic performance disadvantages for swiping. These findings indicate that SMAAT can support mobile survey interactions on participants’ own devices and provide a solid foundation for upcoming longitudinal EMA research that will more fully exploit the platform’s notification, sensing, and analytics capabilities. More broadly, this work contributes to the growing infrastructure for digital health research by providing an accessible platform for deploying EMA studies in behavioral and health science contexts.

## Abbreviations

EMA: ecological momentary assessment
GDPR: General Data Protection Regulation
SMAAT: sensor-based mobile app for assessment and tracking
SUS: System Usability Scale
UEQ-S: short version of User Experience Questionnaire
